# Should We Just Prescribe? Ethical Considerations When Using Antidepressants and Benzodiazepines For Emotional Distress

**DOI:** 10.1007/s11673-025-10437-4

**Published:** 2025-09-15

**Authors:** G. García-Calderó, S. Peregalli Politi

**Affiliations:** 1 Institute for Biomedical Ethics (IBMB), University of Basel, Bernoullistrasse 28, 4056 Basel, Switzerland; 2 Child and Adolescent Psychiatry Clinical Services, Geneva University Hospital, Rue Gabrielle-Perret-Gentil 4, 1205 Geneva, Switzerland

**Keywords:** Antidepressants, Benzodiazepines, Emotional distress, Medicalization, Primary Healthcare, Bioethics

## Abstract

Prescribing antidepressants and benzodiazepines for patients with emotional distress is a common practice in primary healthcare that raises certain ethical questions. This paper has three aims. First, to describe the motivations that lead general practitioners to prescribe antidepressants and benzodiazepines in these cases. Second, to reflect on the ethical implications of such prescriptions based on the four principles of biomedical ethics defined by Beauchamp and Childress (autonomy, nonmaleficence, beneficence, and justice). Finally, to propose some recommendations for the mitigation of the medicalization of emotional distress in primary healthcare. Results show that general practitioners seek to alleviate patients’ suffering but their prescribing decisions are influenced by some uncertainties in clinical judgement as well as by systemic factors (patients’ pressures, time constraints, and unawareness of resources). Ethical issues arise in relation to the potential for dependence, the questionable long-term benefit of prescriptions, the uncritical fulfillment of patients’ expectations, and the impediment to address underlying social issues or to develop patients’ capabilities. Clinical consultation should be founded on effective communication between doctors and patients and a holistic care approach that acknowledges the psychological, social, and existential dimensions should replace a merely symptomatic approach. Some strategies to mitigate medicalization are proposed: the promotion of regular monitoring visits with patients and multidisciplinary collaboration, the enhancement of physicians’ knowledge about non-pharmacological interventions, as well as the establishment of an evidence-base for the effectiveness of these drugs in the primary healthcare setting.

## Introduction

In many Western culture countries, General Practitioners (GPs) are accountable for a vast percentage of antidepressant and benzodiazepine prescriptions, which are the most common drugs prescribed by GPs (Siriwardena 2010). In Norway, for instance, they prescribe the 73 per cent of antidepressants consumed by the general population (Svensson et al. 2019). A study conducted in the region of Catalonia, reported that between 2 and 42 per cent of primary care patients are treated with psychotropic drugs (antidepressants, anxiolytics, antipsychotics, and hypnotic–sedatives) (Rubio-Valera et al. 2012). Intuitively, one would expect such prescriptions to result from the diagnosis of some disease for which such drugs are indicated. However, research attests that a significant proportion of patients have been prescribed with antidepressants without having a mental disorder: according to Demyttenaere et al. (2008) almost 55 per cent of patients seeking help for emotional problems in six European countries were prescribed with antidepressants. Antidepressants are commonly prescribed for variable combinations of physical symptoms and psychological distress (Mercier et al. 2011).

The fact that this type of prescribing occurs mainly in primary healthcare could be explained due to the closeness of this medical field to people’s everyday life and to the strong interrelation between the patient’s health and their personal, social, and environmental circumstances (Zurro and Pérez 1986). Prescribing these drugs when the patient is not affected by any disease entails some health-related and ethical questions. In the Health Plan for 2021–2025 approved by the Government of Catalonia, the use of psychotropic drugs for other situations outside their medical indications is described as a type of medicalization of daily life, in which pharmacology is used to respond to life problems or emotional states (Generalitat de Catalunya. Departament de Salut 2021a). Medicalization, a term coined in 1975 (Illich 1975), refers to the definition of life problems in medical terms and to the application of medical interventions to treat them (Conrad 1992). In the context of modern Western societies, prescribing psychotropic drugs to treat emotional distress is considered as one of the main forms of medicalization (Conrad 1992; Horwitz and Wakefield 2007; Parens 2013; van Dijk et al. 2022). By the end of the twentieth century, the Hastings Centre Report “The goals of medicine: Setting new priorities” (Callahan 1996) portrayed medicalization of daily life as a trait of contemporary medicine and posed the question of the appropriateness of treating unhappiness and daily-life or existential anxieties through drugs.

Substantial bibliography has been developed during the last decades in which medicalization of states of mind has been related to the pharmaceutical industry’s commercial goals. Works such as “The antidepressant era” (Healy 2003), show scepticism about the therapeutic uses stated by antidepressants’ marketers, claiming that these were defined in such a way that many normal states of mind could be included, increasing consequently, the number of potential consumers. This paper will not concentrate on this aspect; rather, it will concentrate on the clinical interaction between GPs and patients.

Based on the evidence of the prescription of antidepressants and benzodiazepines’ for emotional distress, this article has three main objectives:

- First, it intends to identify, describe, and classify the factors that lead GPs to prescribe antidepressants and benzodiazepines for cases of emotional distress.
- Second, it aims at analysing the ethical issues related to this intervention.
- Third, it aims to reflect upon ways to avoid medicalization of emotional distress in primary healthcare.

Hence, this paper is divided in two main parts. The first part is a literature review that focuses on the factors that influence prescribing. The review has been conducted through PubMed, Scopus, and Google Scholar using the PICO strategy (Santos et al. 2007).

The second part offers a bioethical reflection that is informed by the four principles of biomedical ethics outlined by Beauchamp and Childress (1994). Additionally, it provides a set of recommendations to mitigate medicalization in accordance with the ethical implications. The bioethical discussion includes substantial insights from the work “For the Patient’s Good” by Pellegrino and Thomasma (1988) and further draws on other documents relevant to the topic, such as the report from the *Hastings Centre* “The goals of medicine: Setting new priorities” (Callahan 1996) and reports from the World Health Organization (WHO) about Mental Health (World Health Organization 2012), and Primary Healthcare (World Health Organization 1978).

## Factors Influencing Prescribing

This section provides an overview of the factors that lead GPs to prescribe antidepressants and benzodiazepines for emotional distress (see table 1). These have been divided into individual or systemic factors, depending on the main influence bearing on the prescribing decision: either different aspects of the physicians’ clinical judgement or external stressors related to society and to the healthcare system. Nevertheless, these distinctions serve mainly as clarification, rather than showing a differentiated influence in practice. GPs’ ultimate decision is shaped by an interplay amongst these factors. The factors presented in this section are integrated later on in the ethical analysis.

**Table 1:** Summary of drivers for prescribing antidepressants and benzodiazepines for emotional distress

**Individual factors**
Uncertainty in clinical discernment and willingness to help
Guidelines and efficacy and safety considerations
Self-developed criteria and role perception
**Systemic factors**
Dealing with patients’ pressures
Workload and limited time
Scarce resources and limited access to psychotherapy

### Individual Factors

#### Uncertainty in Clinical Discernment and Willingness to Help

The essential criterion to assess if prescribing a particular drug is clinically justified, is the correspondence between the malady from which the patient is suffering and the therapeutic indication of the drug (e.g. Major Depression Disorder—MDD— for antidepressants or Generalized Anxiety Disorder—GAD—for benzodiazepines).

However, the fact that GPs often find themselves in the situation of being consulted by their patients about the effects of personal and social problems creates challenges in diagnosing accurately and distinguishing the need for treatment for depression from other approaches to life problems (Macdonald et al., 2009). Doubts regarding the origin of depressive symptoms, especially their potential association with social circumstances, complicate the clinical judgement of whether to prescribe antidepressants:

… I think for me what is the big dilemma in managing these sort of conditions in general practice is how many of them are simply sad people, with sad lives, coping with difficult life events, which will just with the passage of time and a bit of understanding work through. Or are they genuine depressive illness[es] in the biological sense that are related to chemical changes in the brain, whatever. That is what I find very difficult … (Hyde et al. 2005, 757)

GPs are divided with regard to the question about the nature of problems they treat with psychotropic drugs. A study that presented the same vignette of a woman with sadness complaints and a challenging personal circumstances to a group of GPs triggered a varied response from these: some pointed out to a depression, some were inclined towards a depression diagnosis but were not sure, and others perceived her situation as part of a life-phase issue (van Dijk et al. 2022). Physicians perceive general practice as a particularly difficult setting to distinguish between medical and socioeconomic factors that originate ill health compared to other healthcare domains: patients usually present milder symptoms and patients’ personal situations come easier into play through long-established contact with physicians (Svensson et al. 2019).

The willingness to help patients goes hand-in-hand with the uncertainty about the best way of addressing these problems of social and other aetiologies in the clinical setting. Even though GPs are aware that frequently complex psychosocial situations underlie the distress of their patients, a BDZ prescription may be perceived as the help they can offer as clinicians:

You have to think that if you were in their situation you would not know what to do either. In this situation this person needs a BZD to give him some support for the things that are unbearable. (S1066). (Anthierens et al. 2007, 216)

According to Sirdifield et al. (2013), prescribing benzodiazepines, which have a rapid effect, can be the consequence of a sense of impotence and deemed the “lesser evil”.

Svensson et al. (2019) point out that doctors usually feel compelled by a moral obligation of helping their patients, which goes further than mere professional compliance. van Dijk et al. (2022) content that psychotropic drugs are perceived by some GPs as a tool within reach to reduce distress, which responds to their moral obligation to alleviate patients’ suffering, which is aligned with the findings of Sirdifield et al. (2013): “We want to help people and make people feel better. So, if we give people something and make them feel better, then everybody seems to be happier” (Sirdifield et al. 2013, 7).

Nevertheless, there are also critical voices amongst GPs who recognize that providing patients with antidepressants may not do any good to them in the long run. This is due to the perception that the root of these emotional states are commonly problems with unsatisfying relationships, work, or life in general, and its management falls outside from medicine’s power (Macdonald et al. 2009). Postponing prescribing and adopting a “wait and see” strategy is an often-reported attitude in the face of cases with an unclear origin and prognosis (Mercier et al. 2011). One study has shown that GPs preferred to listen to patients before prescribing, especially those presenting mild to moderate forms of depression: a period of “wait and see” would allow the remission of depressive symptoms for those patients who were especially in need to talk to someone (Johnson et al. 2017). Hyde et al. (2005) stress as well that to wait and see was the preferred option in cases of uncertainty, as it afforded to determine if the condition was self-limiting. Some physicians reported posing a series of clinical questions (e.g.: duration, severity … etc.) with the objective of discarding other pathologies which could be the origin of the symptoms and identifying classical physiological manifestations of depression, such as sleep discontinuation and appetite reduction (Hyde et al., 2005).

Notwithstanding, in cases in which symptoms were regarded as severe and persistent, antidepressants would be prescribed at the first visit. van Dijk et al. (2022) argue that the willingness to do “something” for the patient disclosed by GPs was a hindrance to adopt a “wait and see” strategy. Remarkably, not all GPs found it important to make a diagnosis. Some felt at ease prescribing antidepressants even without a clear diagnosis, given the perceived efficacy of these drugs for a variety of conditions (Hyde et al. 2005). This will be further explored in the next section.

#### Guidelines and Efficacy and Safety Considerations

The latest developed antidepressants, selective serotonin reuptake inhibitors (SSRIs), have shown to cause fewer adverse effects than the classical tricyclic antidepressants (TCAs) in terms of safety after overdose, drop-out rates, tolerability, and cardiac side-effects (Wang et al. 2018). The improved tolerability of SSRIs compared to TCAs has not only been associated with a stronger safety perception by GPs (Mercier et al. 2011) but also, with the perception of having a milder effect, which would justify their use for milder symptoms (Macdonald et al., 2009). Johnson et al. (2017) refer to GPs prescribing confidently SSRIs for distressed patients with depression symptoms, provided that these drugs are deemed safe and effective. However, GPs did not consider long medication continuation as problematic and failed to conduct medication reviews proactively driving the increase in antidepressants’ prescriptions volume (Johnson et al., 2017).

With regard to BDZ, GPs were generally aware of their short-term use indication due to the risk of dependency. Some GPs reported being especially cautious about starting a BDZ prescription and warned their patients that the prescription would only last for a short period of time. In this sense, prescribing BDZ to patients with recurrent anxiety crises was avoided (Anthierens et al. 2007). Informing patients about the short duration of treatment and warning them about dependence development when prescribing BDZ was reported in other studies as well (Parr et al. 2006; Sirdifield et al. 2013):

I actually tell people when I am giving them a short course say for a crisis, that I only want you to take these for 4 or 5 days and then throw the rest of them away because they are habit forming. (Sirdifield et al. 2013, 10)

Regarding the use of diagnostic manuals, GPs’ opinions are varied. Some deploy the DSM manual as well as alternative depression rating scales for guidance in prescribing (Johnson et al. 2017; van Dijk et al. 2022). By contrast, other GPs express scepticism regarding the reliability and usefulness of DSM-IV for the assessment of patients (Mercier et al. 2011) or refrain from using manuals due to their lack of a social domain (Johnson et al. 2017). It is noteworthy, that Munoz-Arroyo et al. (2006) did not find any link between the increased prescription rate of antidepressants and a rise in depression diagnosis. Hence, they argue that one plausible explanation for the high antidepressants’ prescribing rate could be the fact that GPs perceive them as safe drugs.

Some GPs do not follow prescribing guidelines for psychotropic drugs because they consider them unhelpful for decision-making in real situations (Mercier et al. 2011). Some physicians hold that these guidelines were mainly designed for psychiatric settings whereas primary healthcare consists in a distinct scenario with distinct requirements (Šubelj et al. 2010). For others, prescribing guidelines were criticized for being primarily directed to detecting symptoms, hence, overlooking underlying causes: “Current prescribing guidelines emphasise the need to treat symptoms of depression, whether or not social or personal factors may have influenced an episode of low mood” (Macdonald et al. 2009, e304). All these factors contribute to the development of variable criteria amongst GPs to discern when to prescribe.

Svensson et al. (2019) found that there was considerable variation in prescribing for equal symptoms between physicians. Despite the fact that certain influencing factors, such as the specific sociodemographic profile of patients (women, older people, and socially deprived individuals being prescribed more often), can account for some of these variations, a further explanation remains elusive, which has been referred to as “diverse prescription habits of physicians” (Svensson et al. 2019). For instance, Johnson et al. (2017) emphasize the influence of prescribing support teams in order to make cost-effective decisions, such as consultations with the in-house pharmacist, rather than national and local guidelines. To these variations in prescribing patterns refers the next sub-section.

#### Self-Developed Criteria and Role Perception

Some GPs reported how at the beginning they used prescribing guidelines but as they gained more experience they became more idiosyncratic and decided treatment according to their perception of side-effects and “beneficial side-effects for the patient” (Johnson et al. 2017). Sirdifield et al. (2013) identified prescribing patterns for BDZ prescriptions according to the physician’s knowledge of the patient and a rather implicit category which they referred to as the “deserving patient.” Patients who have substance addictions are generally considered non-deserving (especially in light of the high risk of addiction associated with BDZ), by contrast, BDZ prescribing is deemed justified for those patients who have to deal with concomitant diseases or face complex problems. Rogers et al. (2007) also allude to the category “deserving patients” as legitimizing prescribing for patients with overwhelming life circumstances. Hyde et al. (2005) point to the consideration of other “non-clinical” indicators by GPs. For instance, being male was deemed a factor rising concern, given that younger male patients in particular, are seen as reluctant consulters, and hence, their consultations need to be taken seriously (Hyde et al. 2005).

Mercier et al. (2011) show a relationship between experience and prescribing according to “gut feeling,” which refers to an inner intuition regarding the appropriateness of a psychotropic drug prescription. van Dijk et al. (2022) also mention this approach, especially with regard to dealing with sadness complaints among young adults. It must be noted that these justifications lack transparency and make it difficult to judge the clinical and ethical appropriateness of the corresponding prescriptions. There are further factors which are relevant for the assessment of prescribing psychotropic drugs. In section"Uncertainty in Clinical Discernment and Willingness to Help"the challenge of distinguishing depression-related symptoms from other personal and social circumstances of patients was identified as a factor that drives prescribing.

In some cases, however, it is evident that the patient is experiencing distress due to a challenging situation and it is precisely the existence of these underlying circumstances that GPs use to justify prescribing psychotropic drugs. Marquina-Márquez et al. (2022) show that for GPs working in a socially deprived neighbourhood, the decision on prescribing BDZ was not about consulting clinical practice guidelines but a social containment measure—they prescribe them “(…) when people are in a situation that causes them stress or anxiety, to stop them from stealing or from going on to other types of drugs” (Marquina-Márquez et al. 2022). Other studies show that prescription decisions are based on limiting the symptoms and therefore, to enable patients’ proper functioning at work, sleeping better, and getting some respite from their problems (Hyde et al. 2005; Parr et al. 2006). In this sense, benzodiazepines and antidepressants are perceived as “enablers”, that, while not resolving patients’ problems, at least reduce pressure and allow them to begin to take control of themselves, which would have been very complicated otherwise (Hyde et al. 2005; Parr et al. 2006). In such circumstances, the causes of depressive symptoms were not considered to be significant; prescribing antidepressants was deemed adequate independently of the cause: “if somebody’s depressed it doesn’t matter what’s caused it, they still need antidepressants …’” (Hyde et al. 2005).

Notwithstanding, there are GPs who are concerned about not addressing the real issues (Rogers et al. 2007) and others who, when they suspect that life circumstances are the root cause of distress, would prefer to pursue alternative approaches of assistance (Hyde et al. 2005). As presented in the former sections, GPs can consider antidepressants as efficient drugs but adopt a “wait and see” strategy to ensure that the problems cannot be addressed through other means. Moreover, general practitioners use a variety of approaches, some GPs reported offering counselling, sometimes even in combination with antidepressants (Hyde et al. 2005). Other physicians afforded time for spontaneous remission, for instance, using medical certificates, or referrals of a different nature (to gyms for exercise, to link-workers for handling financial hardship, or to libraries for bibliotherapy) (Johnson et al. 2017).

Apart from employing distinct criteria to evaluate the need for prescribing, GPs also perceive their social function differently. van Dijk et al. (2022) describe three profiles to classify the perceived roles of GPs in relation to their patients’ complaints of sadness,: “experts” are those who perceive their patients’ psychological suffering as part of their competence and prescribe when they deem it necessary; “societal GPs,” believe their role to be similar to that of a counsellor with whom patients can discuss their problems and difficulties; and “fast referrers,” are those who do not consider handling their patients’ psychological complaints as their responsibility or who feel incompetent to do so and refer them to a specialist. These differing interpretations of roles, represent different perspectives on the place of physicians in society, which call for further examination.

### Systemic Factors

Medicine is not only determined by its own principles and standards; it is also shaped by society’s values (Callahan 1996). Furthermore, despite the fact that GPs try to exercise caution when prescribing benzodiazepines and prevent their prolonged use, there are certain contributing factors that can influence the physician’s prescribing criteria, such as the difficulty of accessing psychotherapy or the pressure from patients (Archer et al. 2024).

#### Dealing with Patient’s Pressures

Patients who attend a GP consultation expect to receive some assistance, which they occasionally imagine as a tangible solution (Johnson et al. 2017), while most of the times they wish it to be any quick solution to their distress. These are attitudes that have been described as influencing prescribing psychotropic drugs (Hedenrud et al. 2013; Svensson et al. 2019). General practitioners notice that some of the patients wish to find an easy solution to complicated problems (Svensson et al. 2019), and this is also the case in patients with milder symptoms, a fact that GPs associate with wider social expectations about the kind of problems that can be addressed through medicine (Johnson et al. 2017). Hedenrud et al. (2013) describe how in some cases, GPs perceive patients as having the believe that they have a right to feel well, which goes beyond the licit wish of feeling better. GPs participating in the study of Marquina-Márquez et al. (2022) allude to a low level of tolerance of physical or emotional distress caused by professional or relationship issues or a poor body image: “People are quick to go to medical practitioners as soon as something in their life bothers them; it’s as if we were living in a society where … suffering is also pathologised (CMH practitioner-10)” (Marquina-Márquez et al. 2022, 6). Moreover, according to GPs from the study of van Dijk et al. (2022), young people’s sadness complaints were related to a high social pressure stemming from high expectations, coming for instance, from social media.

Although not all patients look for a drug prescription when searching for care (Hedenrud et al. 2013), BDZs have a fast effect that allows them to effectively meet patient’s demands, and so GPs perceive them sometimes as the “easiest choice for people to feel best quickly” (Sirdifield et al. 2013, 9). Some GPs have contended that the use of psychotropic drug medication is more accepted in current societal norms, which may increase the likelihood of patients initiating treatment (Hedenrud et al. 2013). Further, GPs have reported cases of patients who did not want any alternative to a benzodiazepine prescription—whether behavioural advice or other non-pharmacological therapies—which led GPs to be more prone to prescribe (Anthierens et al. 2007). However, Sirdifield et al. (2013) also stress that many times what most influences GPs prescribing attitude is not the pressure that patients exercise but rather the assumptions GPs make about their patients’ expectations. Commonly, GPs acted according to what they thought were patients’ wishes instead of directly trying to clarify them.

Nonetheless, patients may present attitudes that make GPs refrain from opting for prescribing. For instance, they oppose an antidepressant prescription from the beginning, then later on agree to start taking it, only to give up after one week, despite the fact of having been warned that it takes around three weeks to actually perceive some effect (Hedenrud et al. 2013). Not all patients are willing to accept the limitations of antidepressants, and some expect them to be the “solution” to their problem, which in the end leads to dissatisfaction with the outcome, and thus, GPs prefer to explore alternatives to prescribing (Hyde et al. 2005). Alternatively, GPs may be influenced by the positive feedback received from their patients. With regard to attitudes towards prescribing BDZ, Anthierens et al. (2007) describe the influence of patients’ satisfaction: “If you do prescribe them a BZD, they are very grateful. They come back to you and they are so happy because they have finally managed to sleep. That is so important, it makes you feel good (S295)” (Anthierens et al. 2007). Moreover, GPs underscored the challenges they encounter when declining well-known patients’ requests (Anthierens et al. 2007). However, Parr et al. (2006) describe the opposing view: how for some GPs the fact of knowing the patients for long enough was a reason for trust and for not prescribing BDZ even when patients request them.

#### Workload and Limited Time

Svensson et al. (2019) consider that workload and time constraints make the discernment of whether the patient has clinical symptoms sufficient to justify the prescribing of a psychotropic drug, less significant in practice. Even if GPs are reluctant to prescribe on the first consultation, time and patient pressures are perceived as pushing GPs in the direction of prescribing (Hyde et al. 2005). In this regard, certain GPs pointed out that psychotropic drugs appeared to them as a quick and accessible way to deal with patients’ emotional distress and as a way of “getting through a day’s work” (Svensson et al. 2019; Hedenrud et al. 2013). For some it constitutes a form of giving at least some response to patients who deserve more attention but cannot receive it because of time constraints (Šubelj et al. 2010). Prescribing BDZs was deemed less time-consuming than searching for a non-pharmacological strategy or offering counselling during the consultation (Anthierens et al. 2007). Moreover, GPs perceived an elevated prescription volume as a consequence of an excessive workload, which included the number of appointments that need to be completed daily and the patient quota (Marquina-Márquez et al. 2022).

#### Scarce Resources and Limited Access to Psychotherapy

Scarce access to psychotherapy in proportion to the volume of demand is a factor that makes GPs consider psychopharmacology as the only remaining solution that responds to their willingness to care for patients’ needs (Anthierens et al. 2007; Mercier et al. 2011; Svensson et al. 2019). In the study conducted by Šubelj et al. (2010), all physicians complained about limited access to mental health workers, especially in the peripheral healthcare regions. However, an exception must be made in the case of Sweden, a country in which by contrast to the general international scenario, there is high availability of psychotherapy in primary care (Svensson et al. 2019). Interestingly, GPs from this study conducted in Sweden preferred the use of psychotherapy rather than psychotropic drugs for mild psychiatric disease, which might point out the influence of actual possibilities in prescribing preferences.

Access is not only limited to scarcity of psychotherapists, economic reasons also play a role: according to GPs, non-pharmacological approaches are too expensive and less accessible for patients with financial hardship (Anthierens et al. 2007; Mercier et al. 2011). In this regard, Peter Conrad, one of the main authors on the phenomenon of medicalization, criticizes managed care plans’ wide coverage of pharmaceutical treatment for emotional problems in contrast to the coverage of psychotherapy, which he contends incites the use of psychotropic drugs in these situations (Conrad 2005). General practitioners’ scepticism regarding the usefulness of psychotherapy, especially for those patients with more prominent life problems, is also a factor limiting patients’ referral to psychotherapy (Anthierens et al. 2007; Mercier et al. 2011). Other GPs have explicitly mentioned the limited evidence regarding the effectiveness of non-pharmacological interventions (Marquina-Márquez et al. 2022).

In the study conducted by Sirdifield et al. (2013), GPs acknowledged their lack of training in the provision of psychological support. Conversely, in the study of Marquina-Márquez et al. (2022) GPs expressed their lack of familiarity with non-pharmacological interventions and their unpreparedness to implement a social approach during the consultations and within their time constraints.

## Consideration of the Ethical Issues

The purpose of this section is to provide an ethical analysis of the prescription of antidepressants and benzodiazepines for emotional distress, integrating the drivers identified in the previous section. We will draw on the four principles of biomedical ethics stated by Beauchamp and Childress (1994)—respect for autonomy, nonmaleficence, beneficence, and justice. According to Beauchamp and Childress (1994), these are principles that derive from common morality that should guide medical practice. In addition, this section intends to provide recommendations to promote an ethical approach to emotional distress in primary healthcare that acknowledges the benefits and risks of prescribing psychotropic drugs. Prior to beginning the analysis, we will briefly recall some relevant aspects of both the clinical interaction in primary healthcare and the concept of mental health, as these constitute the backdrop of our reflection.

Modern medicine is oriented towards patient-centred care, in which “patients are known as persons in context of their own social worlds, listened to, informed, respected, and involved in their care—and their wishes are honoured (but not mindlessly enacted) during their health care journey” (Epstein and Street 2011, 100). Patient-centred care can adopt a variety of approaches and is characterized mainly by communication, partnership, and health-promotion (Constand et al. 2014). Communication in particular, has been associated directly and indirectly with an improvement in health outcomes (Street et al. 2009). The Declaration of Alma-Ata (World Health Organization 1978)—which outlines the main conclusions and objectives of the International Conference on Primary Health Care—stresses how Primary Health Care should promote community and individual self-reliance and participation in healthcare, and reaffirms that health is an holistic concept that involves physical, mental, and social well-being.

According to the World Health Organization, mental health is “a state of well-being in which the individual realises his or her own abilities, can cope with the normal stresses of life, can work productively and fruitfully, and is able to make a contribution to his or her community” (World Health Organization 2005, 12). The WHO states as well that psychological well-being is influenced by three main determinants:

- individual attributes and behaviours (such as low self-esteem or communication difficulties).
- social and economic circumstances (such as loneliness or family conflict).
- environmental factors (such as poor access to basic services).

Worsened mental health traits such as sadness, tiredness, hopelessness, helplessness, or fear about the future, which cause mental suffering, can derive from any of these determinants (World Health Organization 2012). Consequently, mental health encompasses many dimensions that need to be considered when assessing the appropriateness of a psychotropic drug prescription.

### In the Light of the Four Principles of Biomedical Ethics

#### Respect for Autonomy

All clinical interactions should strive to respect patients’ autonomy; this implies on the one hand acknowledging the right of patients to act according to their values and beliefs and on the other hand to strengthen patients’ capacities for autonomous choice while reducing the influence of disrupting factors (Beauchamp and Childress 1994). Respect for autonomy does not imply following blindly patients’ desires when these contradict what evidence-based medicine indicates (for instance, prescribing a particular drug, be it psychotropic drug, antibiotic, or any other, when this one will not produce benefits or entail some harm). Respect for autonomy is an essential part of patient-centred medicine, as it acknowledges that the desired outcome is one that is both meaningful and valuable for the patient and meets the standard of care. Thus, it brings together the populations’ perspective of evidence and the individual perspective of the patient’s needs and values (Epstein and Street, 2011).

In the previous section it has been shown that GPs frequently feel compelled to prescribe due to the expectations and pressures of their patients to receive a prescription to alleviate their distress. However, to acknowledge patients’ autonomy physicians need not prescribe in accordance with the patient’s preferences; rather, they should allow the patient to become an active agent in healthcare decisions. And in order to involve patients in healthcare choices, patients need to be informed: informed consent constitutes the standard practice of respect for autonomy and requires disclosure and understanding (Beauchamp and Childress 1994).

In the first place, physicians should facilitate patients’ expression of their priorities. This can be achieved by actively probing alongside the prudent anamnesis that many GPs reported conducting prior to prescribing. GPs would be able to understand the values and beliefs of patients and identify any potential misconceptions or biased subjective evaluations. For example, the widespread belief that there is no alternative to a benzodiazepine prescription to reduce their distress or that a prescription will resolve their problem (Sirdifield et al. 2017). The physician will need to disclose all the information that a reasonable person would need to know in order to judge the medical intervention and tailor it to the informational needs of individual patients to ensure that the patient understands the central information (Beauchamp and Childress 1994). This is important in both cases, when physicians propose an alternative approach such as psychotherapy and decline to prescribe, as well as when they believe that a prescription would be beneficial.

Patients might be reticent to accept any of these proposals due to a stigmatized perception of psychotropic drug use or flawed views regarding the efficacy of psychotherapy. Patients’ autonomous choice is impaired when their understanding of their health status and of available healthcare solutions is mistaken or when their expectations do not correspond with the available possibilities. Therefore, GPs should strive to provide a comprehensive explanation of the factors that contribute to patients’ emotional distress. This should include pointing out the influences that go beyond the medical field, the limited evidence for drugs’ efficacy outside of specific diagnosis, and the limits of the best-considered approach for that case.

As far as respect for autonomy is concerned, academic debates have regarded the two main approaches to emotional distress—psychotherapy and psychotropic drug prescribing—as contributing differently to autonomy. Already in 1972, Gerald L. Klerman described two main moral positions regarding the use of psychotropic drugs for dealing with mental suffering: “pharmacological Calvinism” and “psychotropic hedonism.” The former one holds that their use is morally wrong regardless of its efficacy, as it leads to the development of dependency. Conversely, the latter compares them to other substances such as tobacco or coffee which are frequently used to deal with stressful events (Klerman 1972). More recently, Parens (2013) has revisited this distinction using the terminology of object-oriented and subject-oriented approach. The stance in favour of the use of psychotropic drugs would prioritize efficiency and consider them as a shortcut to relieve people from their suffering, while the stance in favour of a word-mediated approach, such as psychotherapy, would prioritize engagement and uphold the capacity of people to listen to reasons and to transform themselves and their contexts (Parens 2013).

Although these distinctions can help to clarify which are the predominant values underpinning each approach, the separation is overly simplistic and does not grasp all the nuances present in the motivations for prescribing identified in the former section. Some GPs reported prescribing, not as a shortcut to relive suffering, but rather as an intermediate step that would allow patients to gain some control over their emotional states and help them to move forward. In some cases, these prescriptions might actually be part of a strategy to help patients to improve their overall well-being by a progressive implementation of supportive measures. This will be further developed in the discussion of the beneficence principle.

#### Nonmaleficence

The principle of nonmaleficence requires intentional avoidance of actions that cause harm and the obligation not to impose risk of harm (Beauchamp and Childress, 1994). In the case of BDZ, the most acknowledged harm amongst GPs was its strong dependence side-effect if taken for longer than recommended. Accordingly, GPs informed their patients about this effect and warned them not to exceed the prescription’s expiration date. The fast effect of these drugs, which provides quick relieve for patients’ uneasiness, was often perceived to outweigh the risks. However, despite the short-term benefit that BDZ might provide, GPs should assess the risk of causing harm associated with impeding the development of patients’ personal and social resources to face similar situations. In the context of complex personal and social background, at least, additional strategies beyond a benzodiazepine prescription should be considered. Regarding antidepressants, SSRIs have proved to be significantly safer than former TCA and, as a result, GPs have reported to prescribe them with a high degree of confidence. Therefore, similar to BDZ, SSRIs can cause harm by impeding the development of patients’ personal and social resources.

Additionally, failing to exercise due care is an act of negligence and constitutes a violation of the nonmaleficence principle (Beauchamp and Childress 1994). What GPs have reported as moving them sometimes to prescribe is precisely avoiding an act of neglect in the context of a potential Major Depression Disorder (Macdonald et al. 2009) or a situation they perceived as critical (Marquina-Márquez et al. 2022). The fear of mental health symptoms worsening because of inaction is a legitimate preoccupation but should be accompanied by a sound clinical judgement rather than a rushed decision. General practitioners are usually hampered in this endeavour: limited time and workload have been described as major systemic factors, further, uncertainty in diagnosis has been referred as a prominent factor influencing GPs assessment. General practitioners reported their lack of solid criteria to judge whether patients were suffering a mental disease or were suffering under the influence of circumstances, or whether the distress is transitory or is at risk of becoming chronic.

As aforementioned, GPs often take a prudence-based approach and prefer to wait and see how the patient evolves with time before prescribing. Nevertheless, when it comes to the clinical decision, it has been shown how criteria can become significantly arbitrary, using implicit categories such as “the deserving patient” or acting according to “gut feeling” resulting ultimately in remarkable variation in GPs responses to the same symptoms. Moreover, prescribing guidelines are not generally approved by all GPs and many find they do not reflect the real-world situations found in primary healthcare, especially regarding the intertwining with social aspects. Consequently, the risk of neglect, or, on the contrary, of over-prescribing is influenced by the limitations of GPs’ in conducting a sound clinical assessment. In some cases, GPs are unable to minimize these risks independently, because solutions may require a multidisciplinary and health-system approach. Nevertheless, GPs claims for targeted information around psychological suffering in the primary healthcare setting, point out to the potential to strengthen education as a solution that would increase confidence and provide resources to manage uncertainty, and hence, minimize the risk of harm.

In terms of neglect, this goes beyond under treatment of mental health conditions. Health is a holistic concept that involves the physical, psychological, social, and existential well-being. If healthcare does not acknowledge these dimensions, it fails short to recognize the patients’ needs and can neglect important aspects that bear on the patients’ overall well-being, regardless of whether they can be directly addressed by the GP. Uneasiness or sadness can well be called “existential suffering” when they derive from deep disorientation in life (Binder 2022). Having a purposeful life is a fundamental need to human beings and a lack of meaning is an important source of psychological distress, which physicians often encounter in consultations (Frankl 2009). It has been argued that the change in cultural patterns, such as the loss of social references regarding existential aspects, for instance, religious leaders, could explain why doctors find themselves having to deal with suffering from this nature (Kaczmarek 2019; van Dijk et al. 2022). According to Binder (2022), such suffering requires a slow, meaning-seeking approach which cannot be fitted in a diagnosis-treatment paradigm. In such cases, it is recommended that physicians make use of their own experience to accompany patients with conversation and to recognize the limits of medicine in giving an answer to it (Callahan 1996).

#### Beneficence

The beneficence principle refers to acting with the goal of promoting the well-being of the patient, and in turn, requires taking positive steps rather than avoiding harm. Pellegrino and Thomasma (1988) offer an understanding of the patient’s good based on different dimensions. The patient’s good refers to (a) the patient’s ultimate good (the above presented need to lead a meaningful life), (b) the patient’s good as a human being (being treated with dignity and being able to develop themselves as persons according to their capacities), (c). the good perceived by the patient (patient’s values and preferences, such as for example, being able to reach a high professional status), and (d) the biomedical good (the medical intervention that is indicated by clinical evidence).

Some of these dimensions have already been briefly presented. The “biomedical good” regarding emotional distress is unclear. Evidence for the efficacy of antidepressants for the variety of symptoms for which these are prescribed—which do not adjust completely to the Major Depression Diagnosis—and the complex psychosocial background which patients usually present, is missing. Moreover, the slow onset of effects can make adherence challenging, and they may not provide the satisfactory management of symptoms which was sought. This absence of evidence undermines their benefit for patients who experience emotional distress, and calls for the development of clinical evidence that embraces off-label prescriptions in primary healthcare and is tailored to this particular setting. Benzodiazepines were generally considered by all GPs as an effective means to provide a relief of symptoms in the short run. What GPs lacked was knowledge about how to integrate other strategies, especially within the limited time of consultations they have.

The “patients’ ultimate good” and “perceived good,” have been mentioned as relevant aspects not to be neglected and as an integral part of respect for autonomy. The beneficence principle demands that GPs actively address these dimensions within the clinical interaction, which could be accomplished through effective communication. Communication is an essential component of the doctor–patient relationship which bears on the overall health of the patient, either directly —through validation of patients’ accounts or listening empathetically patient’s psychological well-being may improve— or indirectly —through a variety of intermediate outcomes that lead to better health such as self-care skills, social support, or commitment to treatment (Street et al. 2009).

General practitioners seemed to comply with the beneficence principle when expressing their perceived duty to do something to alleviate their patient’s distress. In this regard, prescribing was often deemed the only possibility within reach. This readiness to act has been presented as opposed to the “wait and see” strategy, which refrains from prescribing to ensure that symptoms do not disappear with time or would be better addressed through alternative means. The “wait and see” strategy constitutes a first step to a clinical approach that tries to foster the patients’ capacities and promote their available resources; in which a pharmacological intervention may or not be necessary, but which would never be used as a unique intervention. Indeed, the “patient’s good as a human being” in line with the WHO understanding of mental health, necessitates that the individual develop his or her own capacities and deal with normal stresses of life. Therefore, helping to develop the patient’s potential is fundamental.

Mental health or psychological well-being makes up an integral part of an individual’s capacity to lead a fulfilling life, including the ability to form and maintain relationships, to study, work or pursue leisure interests, and to make day-to-day decisions about educational, employment, housing or other choices. (World Health Organization 2012, 2)

In section"Dealing with Patient’s Pressures", it has been shown how some patients prefer to receive a quick and uncomplicated response to their distress and how such desires can turn into pressures towards doctors, who end up prescribing. Prescribing that follows this logic, could conflict with the beneficence principle. Many of the factors contributing to mental health according to WHO, such as low self-esteem, communication difficulties, family conflict, or loneliness —all of which could underlie the patient’s emotional distress—are aspects that require some time to be adequately addressed. In this sense, if the good perceived by the patient is equivalent to receiving a quick solution to their suffering, perhaps with no compromise or with minimal effort, this would be in direct opposition to the time and effort that are necessary to address these factors.

Further, the beneficence principle can be conceived as providing the help that is appropriate for the nature of the problem. Prescribing could oppose the beneficence principle in some cases, if it used as the main response. From a psychological perspective, Binder (2022) points out that in our culture unpleasant or painful situations are commonly explained through language of illness and bodily disorders: experiences of sorrow or melancholy are labelled as depression and fear and failure are described as anxiety. He holds that these states should rather be considered as messengers from reality that require our attention, as negative emotions that are part of life, and thus, we should endeavour to address in a compassionate and accepting manner.

In a similar way, Horwitz and Wakefield (2007) argue that the experience of sadness was regarded in the past more as an unavoidable aspect of life or even as a relevant state for other purposes, in contrast to the current cultural trends. For instance, they put forward an understanding of sadness—when this is not part of a psychiatric condition—as an emotional state intended to bring attention to a human need or aspiration that is not being appropriately met (Horwitz and Wakefield 2007). Such understandings open the horizon to other ways of addressing psychological suffering beyond pharmacological intervention. Certainly, these can entail the integration of other professionals and rely on the patients’ social networks. But precisely such an approach is in the heart of the Alma-Ata Declaration (World Health Organization 1978), which envisions primary healthcare as involving different professionals and the community. These aspects will be further explored in what follows, under the “justice” principle.

#### Justice

Beauchamp and Childress (1994) present the formal principle of justice as giving equally to those who are equal and unequally to those who are unequal. Such an abstract statement requires further specification, and, thus, “need” is said to constitute the moral justification for distribution. The “capabilities theory” developed by Martha Nussbaum (2011) states that humans have a basic need to accomplish capabilities and these are opportunities to reach states of effective functioning and well-being. Amongst the list of ten capabilities, Nussbaum presents one capability named “emotions”: “being able to have emotional attachments to persons and things (…) without having one’s emotional development blunted by fear or anxiety” (Nussbaum 2011, 33–34). Fostering capabilities such as this one, is a morally relevant task.

A study conducted by Durà-Vilà et al. (2011) concluded that those with a lower socioeconomic status were more likely to consider sadness as an illness than those belonging to a higher status. The study argues that people who have more resources of any type (personal, intellectual, social, or economic) have more confidence in the possibility to manage life problems without recurring to a clinical response, whereas people with fewer resources are rather more prone to take on a sick role. These findings are relevant to the moral assessment about prescribing in cases of emotional distress. If patients who turn to primary healthcare with these complaints are those who find it difficult to deal with their situation through alternative means, a straightforward prescription would hamper the development of their inner capabilities and the strengthening of their social resources. Consequently, the principle of justice requires that GPs avoid perpetuating social inequalities by offering individuals who live complex socioeconomic situations alternative approaches that are not exclusively reliant on pharmacology.

Crawford (1980) already reported more than four decades ago, that by treating individual problems which may have its roots in societal issues, medicine covers up the original problem and leaves it unresolved while it continues causing distress. Peter Conrad referred to it as a decontextualization of social problems (Conrad 1992, 223). Therefore, if the psychological suffering that patients experience is caused essentially by their socio-economic circumstances, using psychotropic drugs as the primary response to it would deflect attention away from the underlying socio-economic problem. In turn, the development of policies that could address such problems that contribute significantly to individuals’ distress may be impeded.

Byung-Chul Han contends that a palliative response to suffering that has social roots acts as a barrier to social revolutions. It eliminates the opportunities for a community critique regarding social problems or injustices, and hence, it results in the preservation of harmful situations (Han 2021). Further, Han holds that the prevalence of complaints regarding depressive mood in modern Western societies may be partially explicated by social factors. The fundamental value of our societies would be performance, which would lead to individuals placing excessive pressure on themselves, and experience a feeling of “never being enough.” This fact would trigger finally deep frustration and self-reproach. The high use of antidepressants would be according to Han, indicative of the intention to preserve productive capacity by eliminating any symptoms of distress that could impede performance (Han 2015).

According to the WHO (2012) the best way to act in the context of psychological distress stemming from individual, social, or environmental determinants is through health promotion: by applying strategies in different domains that prevent harm brought about by adverse factors. So, for example, a poor work-life balance, in which a person spends an excessive amount of time working compared to the time he or she has for leisure and rest, can ultimately cause stress and anxiety. The proposed strategies to address psychological distress deriving from this cause would require a more comprehensive approach than a prompt response in the clinical interaction and would require collaboration with other sectors —e.g.: education, housing, employment, social welfare, etc. (World Health Organization 2012). The Hastings Centre Report (Callahan 1996) emphasizes that doctors are in a privileged position to orient patients. They can attain a comprehensive understanding of the patients’ circumstances and the underlying cause of their distress, and they can take appropriate action to direct them to the most suitable professional or social agency according to the nature of the problem.

### Recommendations Based on Ethical Implications

This section aims to provide a brief outline of possible approaches to avoid unnecessary prescriptions and promote well-being, in accordance with the analysis we have conducted on the ethical issues associated with the prescribing of antidepressants and benzodiazepines in primary healthcare for emotional distress.

Ultimately, patient-centred care should strive to transcend the mere alleviation of symptoms and integrate a comprehensive understanding of the patient’s well-being. This entails the acknowledgment of the interconnections between medical outcomes and the existential, psychological, and social dimensions of the human being. Therefore, the initial step in addressing patients’ emotional distress, which is usually influenced by a combination of these factors, is to acknowledge them. Subsequently, GPs should attempt to find the most suitable approaches for addressing these dimensions, which necessitates avoiding the neglect of potential existential suffering from patients, lack of certain capabilities or autonomous skills, or deficiencies in the socioeconomic level.

The key to finding the most appropriate approach is effective communication with the patient. In this process, the patient can explain all the relevant aspects affecting his/her situation and express his/her wishes. The clinician validates the patient’s perception through empathic listening and tries to build a trust relationship, which will facilitate the pursuit of a solution that integrates the patient’s values, clinically relevant aspects, and accessible resources is sought. The initial section’s inquiry into GPs motivations to prescribe showed that clinical judgement was hampered by a variety of deficiencies: the challenges in assessing mental health symptoms, the uncertainty about the efficacy of antidepressants for diverse cases of low mood, and the lack of guidelines that acknowledge the challenges of primary healthcare when dealing with psychological suffering.

As far as clinical judgement is concerned, the following actions could improve GPs insights:

- Enhance the evidence-base of the efficacy of antidepressants and benzodiazepines in patients who do not fully meet the criteria for depression or generalized anxiety disorder but present some related symptoms by promoting research in primary healthcare settings. The stronger the evidence-base that physicians possess, the less arbitrary their judgment will need to be.
- Develop guidelines to assess the need to prescribe antidepressants and benzodiazepines that emphasize thorough communication with the patient. These guidelines should include aspects which make prescribing these drugs more ethically problematic, such as the potential misconceptions and stigmatized views from patients, the unrealistic expectations, the existence of temporary challenging situations and patients who are experiencing socioeconomic hardship. The more nuanced the guide, the more tailored it will be for the varied situations that are given in primary healthcare.
- Foster clinicians’ opportunity for exchange of clinical experiences. One of the main critiques for existing manuals and guidelines is that they are not adaptable to real-life situations encountered by GPs. Peer experience, when shared in an open and constructive environment, is a powerful tool to expand one’s resources for assessment and learn from first-hand situations new insights that can illuminate one’s own challenges.

In the first section it has been shown as well, that systemic factors play an important role in physicians’ attitudes towards prescribing. Physicians felt pressured by time available and patients’ expectations. Further factors that led GPs to prescribe were scarce access to psychotherapy, doubts about its efficacy and appropriateness for certain type of patients, and a lack of awareness of alternative socially oriented approaches. While some of these are complex issues that require a deeper reform of the healthcare system, there are some possibilities that could help navigate these challenges:

- Provide more formation to GPs regarding non-pharmacological approaches to deal with emotional distress at the community level, adapted to GPs availability and time possibilities. This was mentioned in one of the studies looking into GPs perceptions on benzodiazepines (Marquina-Márquez et al. 2022), and is an interesting approach that would expand GPs’ capacity of judgement by becoming more knowledgeable about referral options and further support.
- Foster multidisciplinary collaboration, for consultation, referral, or other support. One of the studies mentioned how inestimable was the help of an in-house pharmacist (Johnson et al., 2017). Alternatively, the Catalan government introduced in 2021 a new professional role for primary healthcare teams which has the specific mission to promote emotional well-being of the community, the so-called “emotional mentors” (Generalitat de Catalunya. Departament de Salut., 2021b). Promoting the empowerment of society to confront stressful life events and providing primary healthcare professionals training in emotional management techniques, are amongst the objectives listed for these mentors.
- Short visits sometimes do not allow GPs and patients to reach a conclusion about the best approach for a particular situation. To facilitate communication, general practitioners could attempt to schedule monitoring visits at shorter intervals. Patients may not receive an immediate remedy; however, by engaging in conversation with the clinician, they can clarify the relevant aspects of their situation and benefit from a non-pharmacological approach that may be more beneficial in the long term.

## Conclusion

This paper had three main objectives. First, it intended to describe the reasons why general practitioners prescribe antidepressants and benzodiazepines for emotional distress. Generally, GPs were moved by beneficence as they endeavoured to alleviate patients’ distress while also acknowledging the risks, such as potential for dependence in the case of benzodiazepines, and the uncertain long-term benefits for both benzodiazepines and antidepressants. While some GPs adopted a “wait and see” strategy prior to prescribing, others followed a more direct prescribing approach. Deficiencies in clinical judgement were nearly ubiquitous: these included challenges in assessing mental health symptoms, lack of appropriate prescribing guidelines, and the development of rather arbitrary criteria for prescribing decisions. Additionally, systemic factors played an essential role, encompassing patient and time pressures, scarce access to psychotherapy, and lack of knowledge about alternative possibilities.

Second, it aimed at analysing the ethical implications of these types of prescriptions. It has been shown how prescribing is not always an unjustified act of medicalization: sometimes it becomes a short-term approach to allow patients to gain a certain level of emotional control. Nevertheless, through Beauchamp and Childress’ (1994) four bioethical principles diverse ethical concerns have been revealed, which result from considering prescribing as an exclusive intervention aimed at alleviating symptoms.

The act of adhering to patients’ requests for psychotropic drug prescriptions does not manifest respect for autonomy. Patients’ demands may be based on misconceptions and cannot be equated with the comprehensive clinical judgement of a physician. The development of patients’ inner and external resources may be impeded by prescribing.

Prescribing could potentially lead to act against the principle of non-maleficence: benzodiazepines are associated to a high risk of dependence, while the benefits of long-term prescriptions’ for both benzodiazepines and antidepressants are uncertain. Further, there is a lack of evidence of antidepressants’ efficacy for psychological suffering that does not comply with the criteria of their therapeutic indications, which hinders GPs’ comprehensive assessment.

With regard to the beneficence principle, the promotion of patients’ well-being does not necessarily entail the immediate alleviation of distress. Emotions such as sadness may require a slow approach that involves actively accompanying the patient.

Finally, justice may be neglected by the fact that patients facing socioeconomic hardship are the ones who usually seek primary healthcare for emotional distress due to a lack of alternative resources to address it. A pure pharmacological approach may deny opportunities to develop capabilities and access helpful social resources. Patient-centred care should embrace the patient’s overall well-being, in a holistic approach that includes the psychological, the social, and the existential human dimensions.

Third, the article pursued the goal of providing some insight into ways to avoid the medicalization of emotional distress. Recommendations provided intend to overcome a mere symptom-alleviating approach through the improvement of GPs resources for dealing with the different dimensions that influence patients’ well-being. In order to avoid arbitrary and simplistic assessments, the enhancement of GPs’ clinical judgement is crucial. Increased frequency of visits over a given period of time might help to foster communication and gain new insights for determining the most suitable approach, despite the limited time that GPs have. Collaboration with the community and other professionals is essential to provide a comprehensive response to the patient’s needs, as outlined in the Alma-Ata Declaration. General practitioners can provide a valuable service by referring their patients to the most appropriate support, which relies upon the trust established between the doctor and the patient.

## Data Availability

Not applicable, no primary data has been collected.
